# Prospective field deployment of Swaasa^®^ AI for community-based screening of tuberculosis and other respiratory diseases in Andhra Pradesh: a feasibility and implementation study

**DOI:** 10.3389/fpubh.2026.1951831

**Published:** 2026-10-29

**Authors:** Vasundhara Nammi, Narayana Rao Sripada, Paramita De, Shubha Deepti Palreddy, Sri Rama Murthy Pullae

**Affiliations:** 1 Medical and Health and Family Welfare Department District Leprosy, AIDS and TB Control Office, East Godavari District, Andhra Pradesh, India; 2 Salcit Technologies, Kothaguda, Hyderabad, India

**Keywords:** artificial intelligence, COPD, diagnostic yield, field deployment, respiratory screening, software as a medical device

## Abstract

**Introduction:**

Tuberculosis (TB) remains a major public health problem in India. In the state of Andhra Pradesh (AP), the burden of TB is compounded by a shortage of specialists and the lack of scalable, real-time community screening tools. We prospectively implemented Swaasa^®^ AI, a software as a medical device (SaMD) product, in a real-world community healthcare setting and evaluated its feasibility and user experience for TB screening, with the additional capability to identify individuals at potential risk of chronic obstructive pulmonary disease (COPD) and asthma. Swaasa^®^ uses machine learning (ML) algorithms to analyze acoustic cough patterns and gather information through guided questions about symptoms. Unlike conventional TB screening, which primarily targets symptomatic individuals, Swaasa^®^ supports symptom-agnostic community screening.

**Methods:**

The platform was deployed in a pilot screening program and integrated into existing healthcare workflows. Trained frontline health workers collected cough recordings using smartphones, and the platform generated automated risk assessments for TB, COPD, and asthma. Over a 6-week deployment, a total of 7,879 individuals from vulnerable populations were screened. Based on risk stratification (Green, Amber, and Red) from Swaasa^®^, high-risk individuals were referred for confirmatory testing, specifically using nucleic acid amplification tests (NAATs) for TB and spirometry for COPD and asthma.

**Results:**

Swaasa^®^ AI identified 780 individuals (~10%) at risk of TB and referred them for confirmatory testing. TB was confirmed in 11 of these individuals, yielding an observed TB yield of 1.41% (11/780). Among symptomatic Swaasa^®^-positive individuals, 7 of 23 were diagnosed with TB, corresponding to an observed yield of 30.4%. Importantly, 36% of the identified TB cases were asymptomatic. In addition to TB, the platform identified that 6.5–9.5% of the population was at risk of COPD and 1.6–1.9% at risk of asthma. Further evaluation using spirometry as part of the clinical assessment for COPD and asthma showed that 95% of high-risk individuals identified by Swaasa^®^ had abnormal findings. The study also demonstrated an excellent system usability score (80 to 93%) and a very high perceived usefulness rating (4.37–5) for the use of Swaasa^®^.

**Conclusion:**

This SaMD model offers a scalable pathway for early identification of respiratory diseases, particularly in populations with limited access to specialist care.

## 1 Introduction

In India, tuberculosis (TB) continues to pose a threat to public health (1–3) are concerned. In Andhra Pradesh (AP), there is a significant need to address delays in diagnosis and missed cases to effectively control the disease. For instance, in East Godavari district, which has a population of nearly 1.83 million, approximately 70% live in rural areas, where access to diagnostic facilities can be very challenging. In addition to TB, chronic respiratory illnesses such as COPD and asthma contribute to the prevalence of respiratory diseases in the region. This burden is further driven by risk factors such as air pollution, tobacco use, household biomass combustion, occupational exposures, and recurrent respiratory infections. Systemic constraints, limited diagnostic infrastructure, shortage of trained personnel, and high patient loads in facilities further compound the challenge. Conventional diagnostic methods, such as chest X-rays and spirometry, are routinely unavailable in rural and semi-urban settings due to their high costs, infrastructure requirements, and operational complexities.

Coughing is a common symptom of respiratory disease and results from the forceful expulsion of air to clear the airways (4). The characteristics of a cough can vary depending on the underlying respiratory condition, and distinct cough sounds have been reported for conditions such as asthma, bronchitis, and pertussis (whooping cough) (5). As coughing is a prominent symptom of pulmonary tuberculosis (PTB), previous studies have also reported characteristic features in the cough sounds of individuals with pulmonary TB that may help distinguish the disease from normal respiratory conditions (6–8). With the rapid advancement of machine learning (ML) and deep learning (DL), increasing efforts have focused on leveraging cough signatures for the detection and assessment of respiratory diseases (9). Swaasa® AI has taken advantage of this opportunity through extensive research and multiple clinical and real-world studies, evaluating cough-based respiratory health assessments across different conditions and populations.

The MedTech AI Innovation Challenge 2025 was an initiative of the Government of Andhra Pradesh (AP) aimed at identifying innovations in AI technology and MedTech solutions suitable for enhancing public healthcare in the state. These innovations underwent a rigorous screening process based on scientific and clinical evaluations, along with their integration into the existing health system. Of the 297 proposals submitted, only 18 startups were selected, including Swaasa® from Salcit Technologies.

An AI-powered software as a medical device (SaMD) product can help address diagnostic gaps by enabling smartphone-based respiratory screening through cough sound analysis and machine learning, supporting scalable and decentralized care in resource-limited settings.

Swaasa® AI, a SaMD product developed by Salcit Technologies, is a non-invasive respiratory screening platform that uses artificial intelligence technology to analyze voluntary cough sounds and identify acoustic biomarkers of respiratory diseases. Designed for rapid and convenient screening and triaging, it helps in the classification of respiratory conditions as normal or abnormal and the early detection of tuberculosis, COVID-19, COPD, and asthma (10–14). The performance of our platform, based on multisite validation studies, is as follows:

Normal vs. Abnormal Detection:

Accuracy 89% | Sensitivity 92% | Specificity 86%.

Pattern Classification:

- Obstructive: AUC 0.8 | Sensitivity 81% | Specificity 94%
- Restrictive: AUC 0.88 | Sensitivity 63% | Specificity 79%
- Normal: AUC 0.79 | Sensitivity 74% | Specificity 85%

Disease-wise:

- Asthma: AUC 0.8 | Sensitivity 83% | Specificity 74%
- COPD: AUC 0.84 | Sensitivity 82% | Specificity 75%
- TB: AUC 0.81 | Sensitivity 80% | Specificity 65%

Thresholds are configurable, allowing optimization for high sensitivity (screening) or balanced accuracy (clinical use).

Clinical validation studies and deployment of Swaasa® in health centers across India have demonstrated promising accuracy in controlled or health facility-based settings.

However, the use of Swaasa® by community health workers as part of routine community screening—including house-to-house visits, screening individuals in their homes, and integrating Swaasa® outputs into existing healthcare workflows—has not yet been fully evaluated in real-world settings. This deployment focused on evaluating the feasibility and implementation of Swaasa® within such a community-based screening workflow.

This implementation study presents findings from the field deployment of Swaasa® AI across two primary health centers (PHCs) in East Godavari district, Andhra Pradesh, conducted by trained Accredited Social Health Activist (ASHA) and Auxiliary Nurse Midwives (ANM) workers. We prospectively implemented Swaasa® AI, a SaMD product, in a real-world community healthcare setting and evaluated the feasibility and experience of using it for screening TB, with the additional capability to identify individuals at potential risk of COPD and asthma.

Previous validation studies primarily evaluated the clinical performance and feasibility of Swaasa® in health centers or study settings. This field-deployment study provides additional evidence on the practical implementation of Swaasa® when used by community health workers under real-world conditions. Specifically, it evaluated the feasibility of house-to-house screening, the ability of community health workers to use the tool as part of their routine activities, the integration of Swaasa® outputs into existing screening and referral workflows, and the operational challenges encountered during community-based deployment. Thus, the study complements previous clinical validation studies by providing implementation and real-world workflow evidence that cannot be fully established through controlled validation studies alone.

## 2 Materials and methods

### 2.1 Study design

A prospective field deployment was conducted over a 6-week period from 18 February 2026 to 7 April 2026. The deployment was conducted across two PHCs, PHC I and PHC II, in East Godavari District, AP, and their associated subcenters. This was a pilot program, conducted under the AP Medtech Innovation Challenge 2025. Figure 1 shows the Swaasa® TB screening workflow.

**Figure 1:**
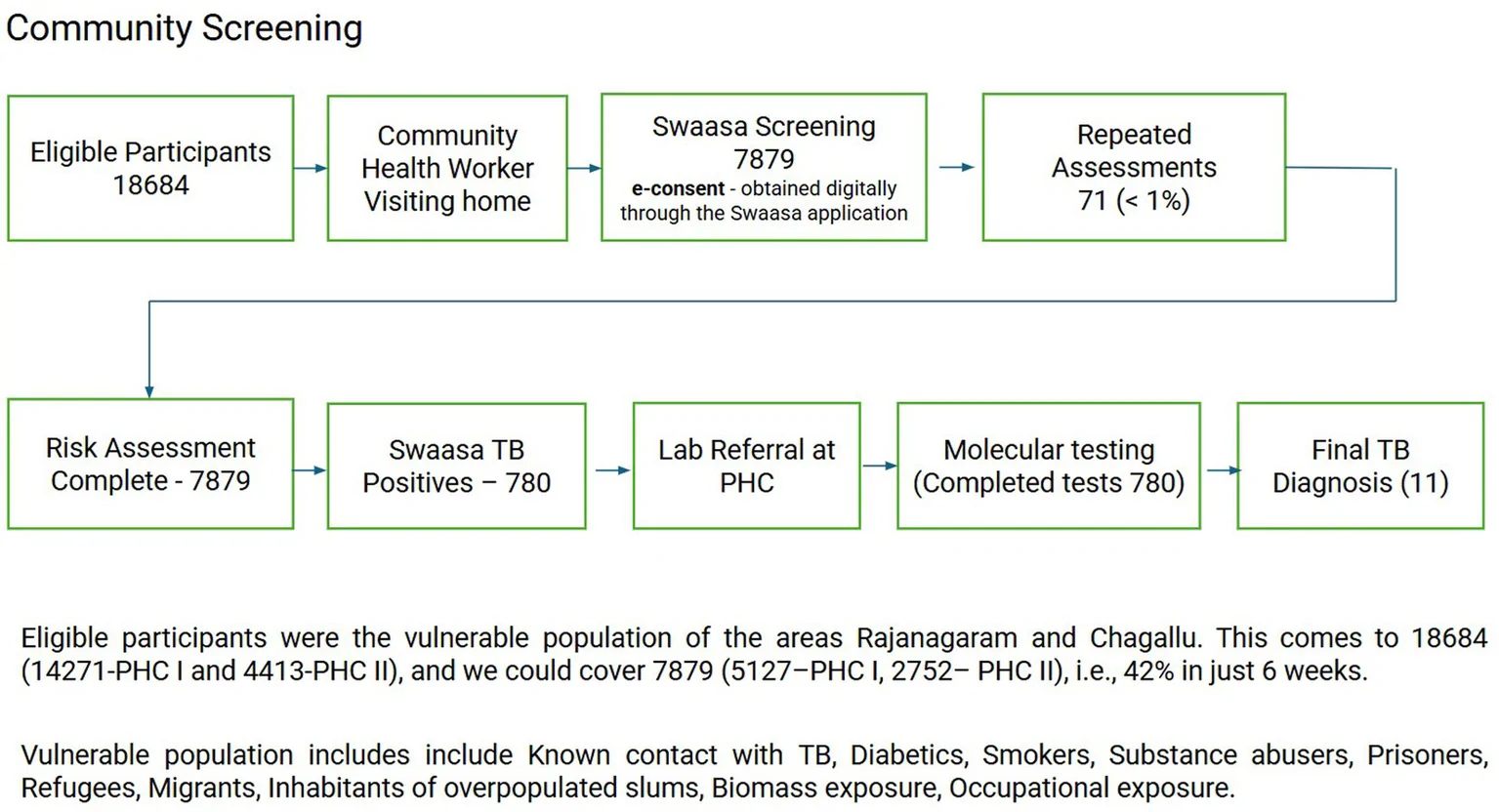
Swaasa^®^ TB Screening workflow.

Frontline health workers, along with Swaasa® field staff, visit community households and conduct assessments using Swaasa®. Based on the report generated by this tool, positive cases are referred for further testing.

Electronic informed consent (e-consent) was obtained from all participants before Swaasa® screening and data collection.

Swaasa® has a defined standard operating procedure (SOP) (referenced and linked here) that outlines the cough-recording procedure, recommended mobile device specifications, and key precautions for health workers to follow while facilitating the recording. Swaasa® runs a preprocessing pipeline that isolates genuine coughs from the recorded audio. The pipeline performs noise suppression, detects cough events with their characteristic air bursts, and applies device-agnostic audio processing, enabling consistent analysis across widely differing recording environments and handset hardware.

### 2.2 Study participants

Eligible participants comprised the vulnerable population residing in the areas of Rajanagaram and Chagallu. The eligible population totaled 18,684 individuals (14,271 in PHC I and 4,413 in PHC II), and we could cover 7,879 (5,127 in PHC I and 2,752 in PHC II) participants, representing 42% coverage in just 6 weeks.

### 2.3 Observed screening and diagnostic yield following deployment

For the final diagnosis, TB status was confirmed using nucleic acid amplification tests (NAATs), which detect the genetic material of *Mycobacterium tuberculosis* and are recommended by the WHO for rapid and accurate TB diagnosis. Respiratory function was assessed using the Spirofy portable spirometer (Cipla ltd), a validated digital spirometry device that measures standard lung function parameters. The observed TB yield and respiratory assessment findings following Swaasa® screening were documented. These findings were also compared descriptively with historical active case finding (ACF) (15) data to provide context for the observed screening yield.

### 2.4 Outcome measures

The analysis was primarily descriptive and performed using predefined numerators and denominators. No formal hypothesis testing or diagnostic accuracy analysis was undertaken. The following were the endpoints:

Primary outcomes: The primary outcomes assessed the real-world deployment of Swaasa® in the community:

1. Screening feasibility and coverage – the proportion of eligible individuals successfully screened and the assessment completion and failure rate.
2. Respiratory risk – the proportion of individuals identified as being at:

- High risk of TB
- High risk of COPD or asthma

1. Observed TB yield – the proportion of individuals identified by Swaasa® who were subsequently confirmed or diagnosed with TB.
2. Observed spirometry concordance – agreement between Swaasa® findings and spirometry results among participants who underwent spirometry.

Secondary outcomes:

1. Usability and acceptability, assessed using the system usability scale (SUS) and percevied usefulness (PU) measures.

### 2.5 Statistical analysis

The total population of the two PHCs (as per the 2011 census) was 32,425. We identified a vulnerable population of 18,684, representing ~57% of the total population. In 6 weeks, we screened 7,879 people, averaging approximately 225 individuals per day.

The sample size was determined based on the number of individuals available for screening during the deployment period. As a result, it represents convenience/operational sampling rather than a statistically powered sample. As there was no randomized or comparison group, and reference-standard testing was not performed systematically on all participants, we have avoided interpreting these outcomes as estimates of sensitivity, specificity, diagnostic accuracy, or clinical efficacy.

## 3 Results

### 3.1 Study enrolment and participant characteristics

Between 18 February and 7 April 2026, 7,879 individuals were screened across the two PHCs. In PHC I and PHC II, 5,127 and 2,752 individuals were screened, respectively. Table 1 presents the male and female proportions.

**Table 1:** Male and Female proportions across the two PHCs.

PHC	Male, *n* (%)	Female, *n* (%)
PHC I (Rajanagaram)	2,338 (45.6%)	2,789 (54.4%)
PHC II (Chagallu)	1,348 (48.9%)	1,404 (51.1%)

Figures 2, 3 show the age-wise distribution and percentage of patients across the two PHCs.

**Figure 2:**
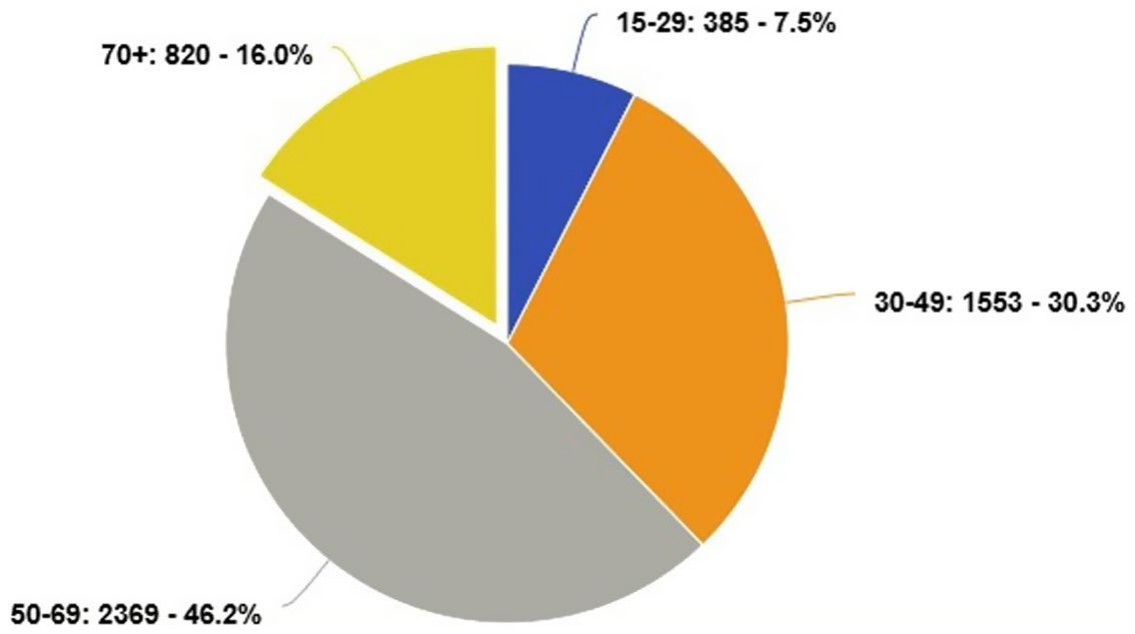
Age-wise distribution of participants in PHC I. The distribution shows that most participants were aged 50–69 years (46.2%), followed by those aged 30–49 years (30.3%), 70 years and above (16.0%), and 15–29 years (7.5%).

**Figure 3:**
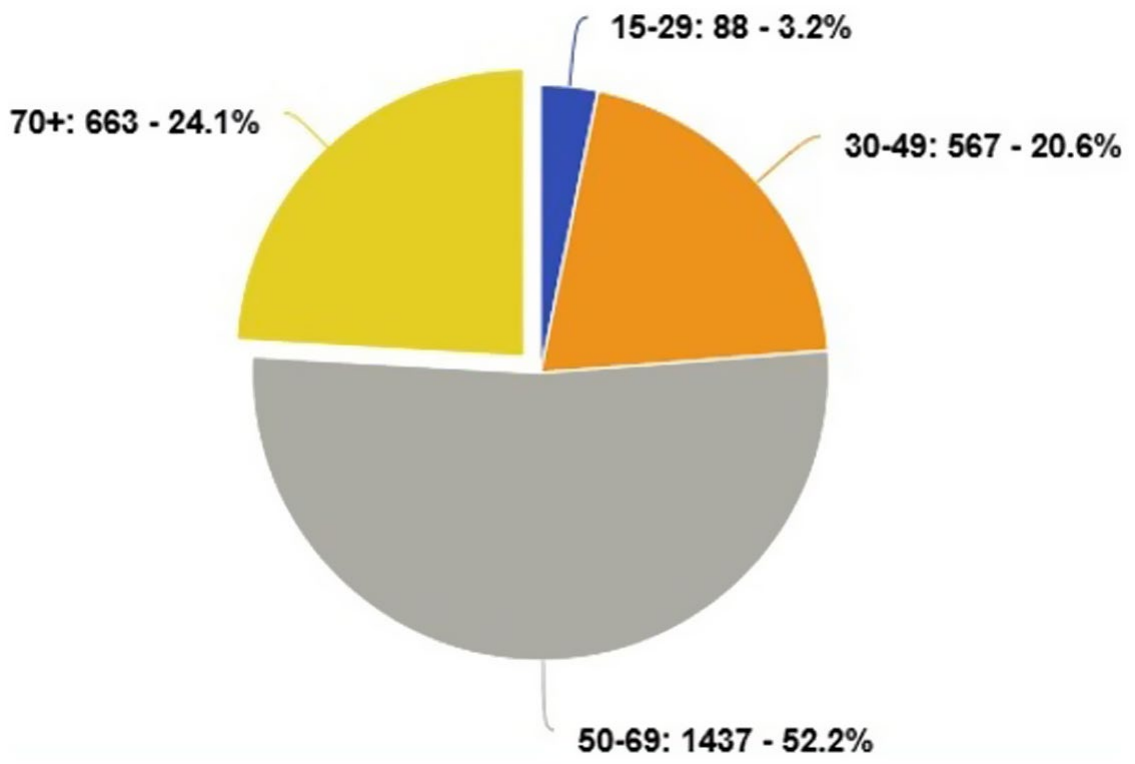
Age-wise distribution of participants in PHC II. The distribution shows that most participants were aged 50–69 years (52.2%), followed by those aged 70 years and above (24.1%), 30–49 years (20.6%), and 15–29 years (3.2%).

### 3.2 Performance analysis

As stated above, 7,879 individuals were screened using Swaasa® AI, of whom 780 were presumed to have TB (symptomatic & asymptomatic) and were referred for TB testing. Among those referred, 11 were confirmed to have tuberculosis. This corresponded to a diagnostic yield of 11/780, 1.41%. The pooled historical ACF data, derived from a different screening cohort and approach, reported a positive yield of 24/1,791 (1.34%). Please note that the ACF data were derived from a historical program that included both community-based screening and outpatient referrals. Importantly, 36% of the detected TB cases were asymptomatic. In addition to TB, the platform identified 6.5–9.5% of the population as being at risk of COPD and 1.6–1.9% as being at risk of asthma. The diagnosis of COPD using spirometry showed good concordance with Swaasa®‘s prediction of COPD risk. In addition, 95% of the tested high-risk participants showed an abnormal spirometry result. Table 2 presents the TB screening outcomes and diagnostic yield of Swaasa® alongside historical ACF data from an earlier period.

**Table 2:** TB screening and diagnostic yield using Swaasa® and historical ACF data.

Metric	Swaasa® Rajanagaram	ACF Rajanagaram	Swaasa® Chagallu	ACF Chagallu
Total screened	5,127	21,462	2,752	6,117
Presumptive TB- positive individuals	550	967	230	824
Presumptive TB yield	10.73%	4.51%	8.36%	13.47%
Referred PTBPs gone for TB Testing	550	967	230	824
TB detected	7	17	4	7
TB Diagnostic yield/Rate of positivity (Confirmed cases/total tested)	1.27% (95% CI – 0.51 to 2.60%)	1.76%	1.74% (95% CI – 0.47 to 4.39%)	0.85%
Case detection rate	0.14% (95% CI –0.05 to 0.28%)	0.08%	0.15% (95% CI – 0.04 to 0.37%)	0.11%

For both Rajanagaram and Chagallu, the NAATs conducted between 2 June 2025 and 17 March 2026 included referrals from both NCD and OPD (PCF) services.

Table 3 presents the symptomatic yield among individuals screened by Swaasa®. Of the 780 individuals identified as positive by Swaasa®, 97% were asymptomatic, leaving 23 individuals with symptoms. Among these 23 symptomatic individuals, seven were diagnosed with TB, corresponding to a symptomatic TB yield of 30.4%. The historical data included PCF and/or more symptom-driven pathways, whereas Swaasa® was deployed for broad, community-based, symptom-agnostic screening.

**Table 3:** Symptomatic TB yield among Swaasa®-positive individuals.

Metric	ACF	Swaasa®
Total Symptomatic	1,791	23
Total TB Detected	24	7
TB Diagnostic yield	1.34%	30% (95% CI: 13.2 to 52.9%)

Table 4 presents the monthly TB screening data and outcomes from Active Case Finding (ACF) activities conducted between November 2025 and January 2026.

**Table 4:** Monthly TB screening data using ACF data from November 2025 to January 2026.

Month/Year	Rajanagaram PHC		Chagallu PHC	
	Tests done	Positive	Tests done	Positive
Nov 25	79	2	32	1
Dec 25	195	3	26	0
Jan 26	11	0	10	0
Total	285	5	68	1

The 4-month average TB diagnostic yield using traditional ACF data was as follows: (i) PHC Rajanagaram – 1.75%, (ii) PHC Chagallu – 1.47%.

Table 5 presents the number of individuals screened for TB and the diagnostic tests performed using Swaasa® during March 2026.

**Table 5:** TB screening and diagnostic testing using Swaasa® – March 2026.

	Swaasa® @Rajanagaram PHC				Swaasa® @Chagallu PHC			
	Screening	Swaasa®-positive	Tests performed	TB-positive	Screening	Swaasa®-positive	Tests performed	TB-positive
Mar 26	2,341	280	429	5	1,488	114	118	4

The average monthly TB diagnostic yield using Swaasa® was *1.7*%.

Overall, Swaasa®‘s ability to identify both symptomatic and asymptomatic individuals may have contributed to the improved case detection observed in community-based screening activities.

### 3.3 Summary: Swaasa®-positive and TB-diagnosed patients

Of the 7,879 individuals screened, 780 (~10%) were identified as Swaasa®-positive (refers to patients identified by Swaasa® as being at medium or high risk of TB (*)). A total of 780 individuals across both PHCs were advised to undergo NAATs. Of these, 11 were confirmed to have TB, of whom ~36% (4 cases) were asymptomatic. These findings highlight Swaasa®‘s ability to detect otherwise missed, early-stage asymptomatic cases. In addition, seven of them belonged to the symptomatic group, reflecting a high yield (~30%), exceeding that of typical ACF-based screening.

#### 3.3.1 (*) Interpretation of TB risk (low/medium/high)

TB disease risk is derived using a proprietary algorithm to calculate a range (low/medium/high). It combines disease-specific symptoms and cough-sound characteristics to generate a risk score. The score is mapped to predefined low-, medium-, and high-risk categories using prespecified thresholds. In this deployment, these categories guided referral for further evaluation and confirmatory testing and were not considered diagnostic.

#### 3.3.2 Demographics of patients diagnosed with TB

Of the 11 TB-positive individuals, 64% (7) were women, while 36% (4) were men. Figure 4 shows the same pattern.

**Figure 4:**
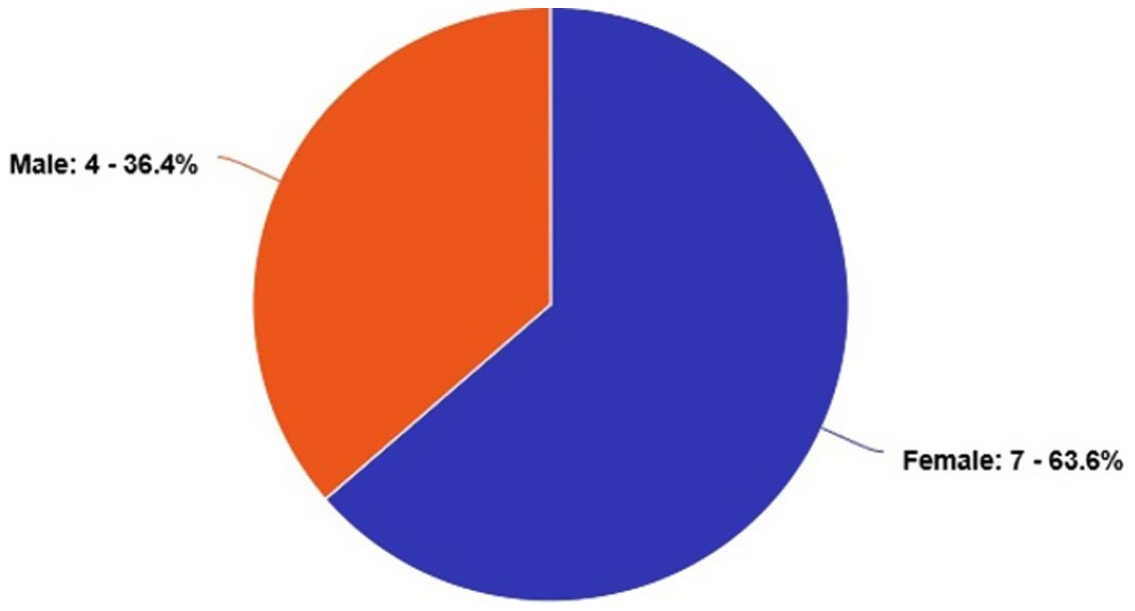
TB-positive patients: Male versus female.

The majority of TB cases were concentrated in the.

50–69-year age group, accounting for ~55% of cases. The age distribution of TB cases is shown in Figure 5. In addition to TB, the platform also screened for COPD, asthma, and overlapping respiratory risks (TB + COPD/asthma).

**Figure 5:**
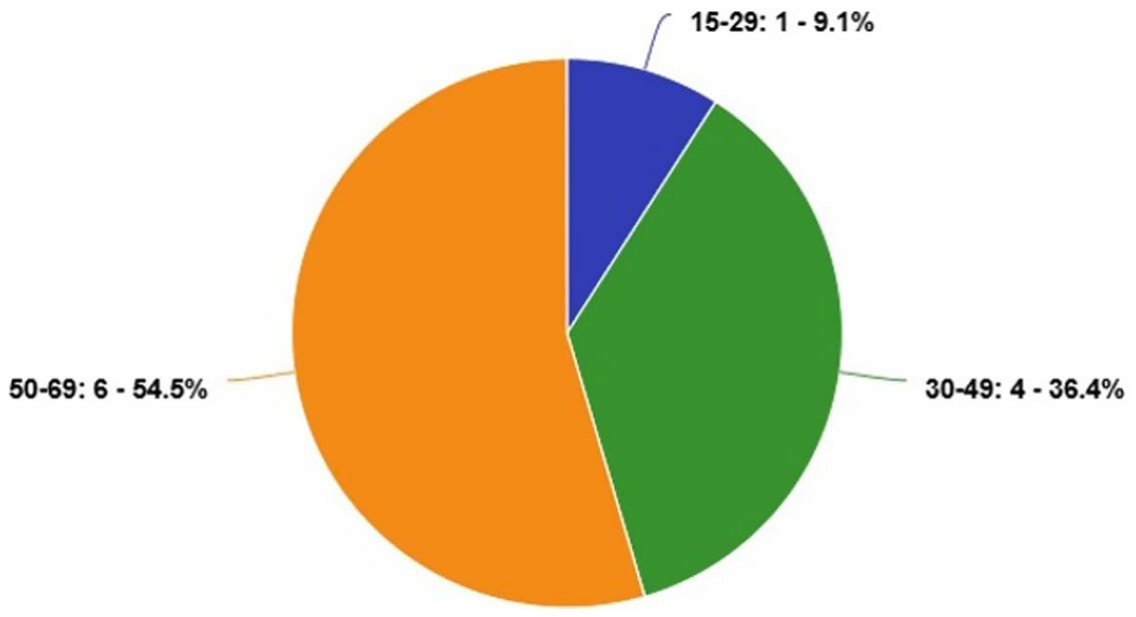
Age distribution of individuals diagnosed with TB.

### 3.4 Participants at risk of COPD

Swaasa® identified that a notable proportion of individuals were at risk of COPD across both locations, with a significant share of these individuals being asymptomatic. Spirometry was performed only in a subset of participants identified as high-risk by Swaasa®, based on the availability and operational feasibility of testing at the participating PHCs. Consequently, spirometry was not performed systematically across all Swaasa-positive or Swaasa-negative participants, and no low-risk comparison group underwent spirometry. The spirometry results therefore represent a follow-up assessment of a selected high-risk subgroup rather than an independent diagnostic validation cohort.

At PHC I, 6.5% (333) of the screened population was identified as being at high risk of COPD. Among these individuals, 52% (173) were asymptomatic.

At PHC I, 58 of the 333 high-risk individuals underwent spirometry testing using Cipla’s Spirofy device. Of these, 54 (93.1%) had abnormal findings, further supporting the accuracy of Swaasa®'s risk classification.

At PHC II, the proportion of individuals identified as being at high risk of COPD was higher, at 9.5% (261) of those screened. Strikingly, 82% (214) of these individuals were asymptomatic, highlighting an even greater gap between actual risk and symptom presentation in this population.

At PHC II, spirometry testing was performed for 102 of the 261 high-risk COPD individuals, with 97 (95.1%) showing abnormal results, indicating strong agreement with Swaasa®‘s predictions.

Across both sites, there was 100% concordance between Swaasa®‘s identification of obstructive respiratory conditions and spirometry-confirmed obstructive patterns, demonstrating its robustness in detecting airflow obstruction. However, the higher prevalence of restrictive patterns observed on spirometry warrants further investigation, particularly as some participants were already receiving treatment for asthma or COPD.

### 3.5 Participants at risk of asthma

Swaasa® was able to screen a measurable proportion of individuals who were at risk of asthma at both locations, demonstrating its ability to flag respiratory risk beyond TB. Among the 7,879 participants screened, 1.9% (97) at PHC I and 1.6% (44) at PHC II were at risk. Although these percentages are relatively small, they are significant in the context of community screening, where asthma often remains underdiagnosed, especially in individuals with mild or unrecognized symptoms.

### 3.6 Overlapping risk (TB with COPD and asthma) analysis

Additional analysis found an overlap in respiratory disease risk within the screened population. It was observed that 2.17% of participants were in the high-risk category for both TB and COPD, whereas 0.85% of the screened population was identified as being at risk of both TB and asthma. Such overlaps are indicative of underlying comorbidities.

Smoking is a common risk factor for both TB and COPD, which, along with its capacity to worsen COPD, causes an increased susceptibility to TB due to a weakened lung immune response.

### 3.7 Usability and acceptability

To assess how user-friendly the device is, a usability evaluation was conducted in a simulated environment using direct observations of operators of the test, followed by a self-administered survey: the SUS. Of the 42 health workers (Auxiliary Nurse Midwives and mid-level health providers (MLHP)) who assisted in the field, 32 participated in this survey/usability study: 20 from Rajanagaram and 12 from Chagallu. Table 6 presents the participants’ roles at both locations. The overall response rate was 73.8%.

**Table 6:** Participant roles at Chagallu and Rajanagaram.

Participant roles	Chagallu	Rajanagaram
Auxiliary nurse midwives (ANM)	8	6
Mid-level health providers (MLHP)	4	8
Community health officer (CHO)	0	6
Total	12	20

#### 3.7.1 System usability scale (SUS)

The SUS is a simple, widely used method to measure how easy a system, like an application or software, is to use. Usability was assessed using a standardized 10-item questionnaire designed to evaluate the perceived usability of a system. Each item is rated on a 5-point Likert scale, ranging from 1 (strongly disagree) to 5 (strongly agree). The SUS combines five positively framed and five negatively framed statements to prevent response bias. The SUS scoring procedure was applied as follows. For the odd-numbered items (Q1, Q3, Q5, Q7, and Q9), 1 was subtracted from the response score. For the even-numbered items (Q2, Q4, Q6, Q8, and Q10), the response score was subtracted from 5. Thus, each item contributed a score between 0 and 4. The scores for all 10 items were then summed, giving a possible total between 0 and 40, and the resulting value was multiplied by 2.5 to obtain the final SUS score on a 0–100 scale, as per standard procedure (16, 17).

In the present assessment, the mean response for each SUS item was first calculated from the available participant responses. For Chagallu, the sum of the adjusted SUS scores was 33.52, resulting in an overall SUS score of 83.8 out of 100. For Rajanagaram, the sum was 37.2, resulting in a SUS score of 92.6 out of 100. These scores indicated good usability in Chagallu and very high usability in Rajanagaram.

For the 12 participants from Chagallu, individual SUS scores were 95, 87.5, 70, 80, 82.5, 82.5, 82.5, 95, 80, 70, 85, and 95. The mean SUS score was 83.8 (SD 8.6). The median score was 82.5, with an interquartile range of 80.0–89.4 and a score range of 70–95. In total, five of the 120 SUS item responses (4.2%) were missing across three participants. Missing responses were assigned a neutral midpoint value of 3 before calculating the SUS scores. For the 20 participants from Rajanagaram, average SUS scores were 97.5, 92.5, 90, 95, 92.5, 95, 90, 92.5, 90, 100, 92.5, 95, 95, 92.5, 92.5, 92.5, 87.5, 90, 95, and 85. The mean SUS score was 92.6 (SD 3.4), with a median of 92.5, an interquartile range of 90–95, and a score range of 85–100. One of the 200 SUS item responses (0.5%) was missing. The missing response was assigned the neutral value of 3 before applying the standard SUS scoring procedure.

The potential for non-response bias was considered low, as only 6 of the 320 possible responses (1.88%) were missing and the missing responses occurred randomly. The potential for social-desirability bias was also considered low, as participants were encouraged to provide honest feedback based on their actual experience, were not prompted to give positive responses, and were not required to provide their identities.

The survey was conducted using a physical questionnaire. The purpose and instructions were clearly explained to all participants, who were given one day to complete the questionnaire. The completed questionnaires were collected on the following day.

#### 3.7.2 Perceived usefulness (PU)

It refers to the extent to which a person believes that using a system will improve their performance or work outcomes. PU (18) was evaluated using six questions that assessed whether the test helped participants complete tasks faster, improve job performance, increase productivity, improve effectiveness, make their work easier, and provide support for their roles. Each question was rated on a 5-point Likert scale, ranging from strongly disagree to strongly agree. The overall mean PU score was calculated by summing the scores across all questions and participants and dividing the total by the number of valid responses.

For Chagallu, the mean PU score was 4.37 (SD 0.49). The median score was 4, with an interquartile range of 1. For Rajanagaram, the mean PU score was 5 (SD 0). The median score was 5, with an interquartile range of 0. These results indicate that participants found the test highly useful at both locations, with higher perceived usefulness observed in Rajanagaram. Table 7 presents the SUS and PU scores recorded at both centers.

**Table 7:** SUS and PU scores at both PHCs.

Metric	PHC Chagallu	PHC Rajanagaram
System usability score (SUS)	83.8	92.6
Perceived usefulness (PU)	4.37	5

#### 3.7.3 Interpretation of the metrics

Usability was measured using the SUS. An SUS score of 68 or higher was considered satisfactory, while a score above 80 indicated very good usability. PU was measured using a 5-point Likert scale based on the technology acceptance model (TAM). Higher average scores indicated greater perceived usefulness.

#### 3.7.4 Key takeaways from the survey/usability study

- Swaasa® SaMD was user-friendly and widely accepted.
- The system was easy to use under field conditions.
- It simplified and accelerated door-to-door screening.
- It enhanced workflow efficiency.
- It saved time and reduced dependence on manual processes.

The usability evaluation included 32 of the 42 frontline health workers; the remaining 10 were unavailable due to operational and personal reasons. Participants were selected from the healthcare workers available during the assessment period, and none had prior experience with Swaasa®. A standardized half-day training was provided at both sites. The questionnaire was self-administered and anonymous. Questions were explained in both English and the local language, and all participants confirmed their understanding before completing the questionnaire.

### 3.8 Key impact

The Swaasa® deployment demonstrated the feasibility of AI-assisted community respiratory screening and identified individuals who may not have been detected through symptom-based screening alone. Of the 780 individuals identified as positive by Swaasa®, 97% were asymptomatic, while 36% of the TB cases identified were asymptomatic. Based on the historical ACF data, the pooled TB positivity was 1.34% (24/1,791), compared to 1.41% (11/780) observed during the Swaasa® deployment. Swaasa® was deployed exclusively through community-based screening, whereas the historical ACF data included a combination of community screening and PCF/outpatient referrals. Given the differences in the two cohorts and screening periods, this comparison should be interpreted as a descriptive observation. Among the 23 symptomatic Swaasa®-positive individuals subsequently evaluated, seven were diagnosed with TB, representing an observed TB yield of 30.4%. Furthermore, 50% of asymptomatic cases had unusual breathing patterns, indicating that Swaasa® has the capability to diagnose other respiratory illnesses. Swaasa® showed good operational efficacy, with a low error rate (< 1%), excellent usability (80 to 93%), and high perceived usefulness (4.37–5).

## 4 Discussion

### 4.1 Principal findings

This pilot deployment should be interpreted as a real-world implementation and feasibility evaluation rather than a diagnostic accuracy study. Swaasa® was deployed as an initial community-level screening and risk stratification tool, with individuals classified as having medium or high TB risk subsequently referred for confirmatory testing. Of the 7,879 individuals screened, 780 were classified as being at medium/high risk of TB. Confirmatory tests were performed for all 780 individuals, resulting in 11 confirmed TB cases. These findings demonstrate the feasibility of integrating AI-assisted, cough-based screening into community and primary-care workflows and its potential to support targeted referral for confirmatory testing. They should not, however, be interpreted as evidence of diagnostic accuracy or population-level effectiveness, as the study did not apply a reference standard to all screened individuals and was not designed to estimate sensitivity or specificity.

In addition to TB risk stratification, the deployment provided an opportunity to identify individuals at potential risk of other respiratory conditions, particularly COPD and asthma. In a subset of individuals identified as being at elevated risk of COPD, subsequent spirometry demonstrated a high proportion of abnormal findings, supporting the potential role of the technology as a tool for prioritizing individuals for further respiratory evaluation.

This field deployment generated additional real-world community data that can support future algorithm refinement and evaluation across diverse community settings.

### 4.2 Limitations

The limitations include the following:

- This was an observational field-deployment study rather than a prospective diagnostic accuracy study with systematic reference-standard testing.
- Confirmatory testing was primarily directed toward individuals identified as being at medium/high risk; therefore, disease status among individuals classified as being at low risk was not systematically established. Consequently, sensitivity, specificity, negative predictive value, and diagnostic accuracy cannot be reliably derived from this dataset.
- The low observed proportion of confirmed TB cases among referred individuals indicates a substantial burden of false-positive or unconfirmed referrals and highlights the importance of evaluating downstream diagnostic capacity and the patient burden.
- The study was restricted to two PHCs in one district and was conducted over a limited deployment period; therefore, the findings may not be generalizable to other populations, districts, seasons, healthcare settings, or implementation environments.
- The comparison with routine ACF-based diagnostic yield should be interpreted cautiously because the available routine NAAT data included referrals from both ACF and OPD/PCF pathways, rather than representing a contemporaneous randomized comparator group.

## 5 Conclusion

The deployment of Swaasa® AI demonstrated the feasibility of using AI-assisted, cough-based screening to identify individuals at elevated risk of TB in a community setting and facilitate targeted referral for confirmatory testing. In addition to TB risk stratification, Swaasa® AI provided an opportunity to identify individuals potentially at risk of COPD and asthma, including those who may not otherwise have entered disease-specific screening pathways.

These findings suggest a potential role for AI-assisted respiratory screening as an initial risk stratification approach within primary healthcare and community settings.

## Regulatory approval and ethical considerations

The government of Andhra Pradesh, through the RITH–HealthTech innovation challenge, granted permission to Salcit technologies Pvt. ltd. to deploy Swaasa® for community-based screening of PTB, COPD, and asthma in east Godavari District through proc. no. E 2764166 /CHFW/NHM/2026 dated 13-02-2026. The approved activities were conducted at PHC Rajanagaram and PHC Chagallu, subject to government conditions on data sharing and confidentiality.

Electronic informed consent was obtained from all participants before initiation of the assessment. Only participants aged 18 years or older were included.

The Swaasa tool is certified under ISO 13485 and has received CDSCO approval for marketing and distribution in India. For data security, privacy, and confidentiality, the platform is certified under ISO/IEC 27001 and ISO 13485 and is compliant with HIPAA requirements. These measures support the protection of participant identifiers and other personal information and ensure adherence to applicable national regulatory and data protection requirements.

## Data Availability

The raw data supporting the conclusions of this article will be made available by the authors, without undue reservation.
