# Supplementary material for: Prospective field deployment of Swaasa® AI for community-based screening of tuberculosis and other respiratory diseases in Andhra Pradesh: a feasibility and implementation study
Source: Front Public Health. 2026 Oct 29;14:1951831. doi: 10.3389/fpubh.2026.1951831 (PMC13755183; doi:10.3389/fpubh.2026.1951831)
Supplement: Supplementary file 1 [file Supplementary_file_1.docx]

The following table presents questionnaires used to assess System Usability Scale (SUS).

| **Num** | **Question** |
| --- | --- |
| Q1 | I think that I would like to use this test frequently. |
| Q2 | I found this test unnecessarily complex. |
| Q3 | I thought the test was easy to use. |
| Q4 | I think that I would need the support of a technical person to use this test. |
| Q5 | I think the various functions in this test were well integrated. |
| Q6 | I thought there was too much inconsistency in this test. |
| Q7 | I would imagine that most people would learn to use this test very quickly. |
| Q8 | I found the test very cumbersome to use. |
| Q9 | I felt very confident using this test. |
| Q10 | I needed to learn a lot of things before I could get going with this test. |

The following table presents the questionnaires used to assess Perceived Usefulness (PU).

| **Num** | **Question** |
| --- | --- |
| Q1 | Using this test in my job would enable me to accomplish tasks more quickly. |
| Q2 | Using this test would improve my job performance. |
| Q3 | Using this test in my job would increase my productivity. |
| Q4 | Using this test would enhance my effectiveness on the job. |
| Q5 | Using this test would make it easier to do my job. |
| Q6 | I would find this test useful in my job. |
